# Editorial: Regulation of Mitochondrial Function on Animal Diseases

**DOI:** 10.3389/fvets.2022.943860

**Published:** 2022-06-17

**Authors:** Hui Zhang, Yung-Fu Chang, Jianzhu Liu

**Affiliations:** ^1^College of Veterinary Medicine, South China Agricultural University, Guangzhou, China; ^2^College of Veterinary Medicine, Cornell University, Ithaca, NY, United States; ^3^College of Veterinary Medicine, Shandong Agricultural University, Taian, China

**Keywords:** regulation of mitochondrial function on animal diseases mitochondria, apoptosis, oxidative stress, energy metabolism, immune response

Mitochondria provide necessary energy for cells and participate in cell differentiation, cell information transmission, cell apoptosis, oxidative stress, and other processes (1). Meanwhile, mitochondria have the ability to regulate cell growth, cell cycles and play an essential role in the regulation of various animal diseases. When mitochondrial dysfunction occurs, it is accompanied by a series of diseases. This topic aims to study the regulatory role of mitochondria in the occurrence of various animal diseases. The 12 papers were collected in this topic, expounded on the role of mitochondria in controlling apoptosis, autophagy, oxidative stress, energy metabolism, signal transduction, and other aspects from different perspectives.

## Mitochondrial Damage and Oxidative Stress

Excessive ROS production can lead to mitochondrial membrane damage. Studies show that methionine-deficient broilers at 42 days of age have apparent kidney and liver damage with the change of mitochondrial structure and lipid metabolic disorders (Song et al.). Therefore, lipid metabolism disorder may be the secondary phenomenon of this pathological change, and mitochondrial damage is the first cause. Mitochondria are also affected by an excess of ROS, eventually leading to oxidative stress. Mitochondrial quality control (MQC) is essential for maintaining mitochondrial function. MQC is a safety mechanism of mitochondria, which can maintain the normal operation of mitochondrial biogenesis, mitochondrial division, mitochondrial fusion, and mitochondrial phagocytosis (2). In the study of vanadium (V) induced cardiac oxidative stress and mitochondrial quality control disorders in ducks, it was found that excessive V could cause cardiac oxidative stress in ducks, and V increased the levels of H_2_O_2_, MDA, and CAT, and the associated mitochondrial damage would also occur, leading to mitochondrial dysfunction (Xiong et al.).

## Mitochondria Regulate Apoptosis and Autophagy

Mitochondria-mediated signal transduction linked to an internal apoptosis pathway. P53 can interact with Bcl2 family members in the outer membrane of mitochondria, catalyze Bak activation and promote transcription-independent activation of Bax. Subsequently, activated Bak and Bax oligomerize in the outer membrane of mitochondria, inducing permeability. Caspase-9 is activated by signal transduction mediated by internal mitochondria, leading to activation of Caspase-3, thereby promoting apoptosis (3, 4). Conversely, Bcl2 inhibits the activation of Bax and Bak, thereby inhibiting apoptosis. Study showed that aflatoxin B1 (AFB1) inhibited the transcription of Bcl2 in broiler kidney tissue, promoted the transcription of P53, Bax, Bak1, CyC, Casp9, and Casp3, and induced renal cell apoptosis through the mitochondrial pathway. These findings suggest that AFB1 may activate mitochondrial-mediated apoptosis pathways (Tao et al.). With the addition of AFB1, it was found that the transcription of Bcl2 was reduced, and the expression levels of Casp3, Casp9, p53, Bak1, and Bax were observed to increase, while the addition of Herba extract (PCPE) had the opposite effect. These results highlight the involvement of AFB1 in mitochondria-related apoptosis (Nabi et al.). In addition, the study on the effects of dioxafimin (DQ) poisoning on mitochondrial apoptosis in duck liver also found that anti-apoptotic genes Parkin and Bcl2 were significantly up-regulated after DQ treatment, suggesting that anti-apoptotic factors play an important role in ducklings during acute DQ poisoning (Chen et al.). Adenosine triphosphate binding box subfamily C member 9 (ABCC9) protein is associated with bioelectrical control. The blocking of the KATP pathway controlled by G protein can significantly inhibit the proliferation and induce apoptosis of glioma and xenograft cells. In the study of down-regulated ABCC9 expression in canine breast cancer, it was found that ABCC9 and ABCC9 mRNA levels on the cell membrane of malignant tissues were significantly reduced, and ABCC9 expression was negatively correlated with cancer grade, and positive cells in the grade III cancer samples basically disappeared. This relationship may be due to the inhibition of KATP channels in cancer tissues (Hao et al.). Excessive V decreased the mRNA levels of Parkin, PINK1, P62, LC3B, and PGC-1α, NRF1, and TFAM, as well as the protein levels of PINK1 and PGC-1α, suggesting that excessive V could lead to biogenic damage of duck heart mitochondria, resulting in the defects of mitotic phagocytosis in myocardial cells. It is speculated that excessive V can seriously damage cardiac myocytes and mediate mitochondrial phagocytosis defects by inhibiting PINK1/Parkin signaling pathway (Xiong et al.).

## Mitochondria are Involved in Energy Metabolism

The long chain enyl-CoA hydrase (OMIM 609015, EC 4.2.1.74), an enzyme that is integrated into the mitochondrial trifunctional proteins, is dysfunctional, and it remains to be seen whether genetic defects cause atypical myopathy in neonatal foals (Sander et al.). Glucose and serum-free fatty acid levels did not alter acute succinic acid (SUC) management, but lactic acid levels were significantly reduced in skeletal muscle. The results showed that SUC's explosive power increased did not depend on glycolysis and β -oxidation of fatty acids. After SUC treatment, ATP increased by increasing electron transport efficiency and oxidative phosphorylation. Moreover, SUC decreased NADH/NAD+ ratio, enhanced complex mitochondrial enzyme, increased creatine kinase (CK) activity, and increased skeletal muscle strength by increasing ATP production. Mitochondrial calcium levels strictly control mitochondrial ATP production, which stimulates complex III and ATP synthase on the electron transport chain to accelerate oxidative phosphorylation, and SUCNR1 activation has been shown to activate calcium signaling (Xu et al.). The proton drive drives the oxygen consumption of mitochondria, which is present in the inner membrane of mitochondria and drives the production of ATP. When the energy requirement of cells increases, the proton drive increases, increasing the oxygen consumption rate (OCR). OCR of vaccinated hens was significantly higher than that of unvaccinated hens, indicating an increase in the oxidative capacity of activated immune cells (Meyer et al.).

## Mitochondria Participate in Antiviral Immunity

Mitochondria, which play a key role in apoptosis, become an important part of antiviral immunity. Novel mitochondrial proteins of mitochondrial antiviral signaling proteins (MAVS), as well as mitochondrial DNA and mitochondrial ROS, produced as sources of hazard-associated molecular patterns (DAMP), will be involved in antiviral immunity. The adaptive development of mitochondrial antiviral proteins in mammalian species was revealed, and the CARD domain of MAVS protein is highly conserved in mammals, which seems to be related to its participation in the adaptive immunity of the organism (Ahmad et al.).

It is also worth mentioning that Gao et al. determined the complete mitochondrial DNA (MT DNA) sequence of fluke lamiformis intermediates based on gene content and genome organization in Yaks of Qinghai-Tibet Plateau for the first time. Furthermore, the activation of TLR1/2/6/9/MyD88/NF-κB and TLR3/TRIF/IRF signal transduction pathways promotes the production of inflammatory factors, which provides a theoretical basis for the control of mastitis caused by *M. bovis* (Yang et al.).

Together, these results represent the latest data on the regulatory role of mitochondrial function in animal diseases. Despite all the existing literature and evidence on this extremely important topic, the papers published in this ebook clearly show mitochondria's role in controlling apoptosis, autophagy, oxidative stress, energy metabolism, signal transduction, and more. By the end of this book, the reader will understand the regulatory roles mitochondria play in animal disease.

## Author Contributions

HZ, Y-FC, and JL wrote the article. All authors contributed to the article and approved the submitted version.

## Conflict of Interest

The authors declare that the research was conducted in the absence of any commercial or financial relationships that could be construed as a potential conflict of interest.

## Publisher's Note

All claims expressed in this article are solely those of the authors and do not necessarily represent those of their affiliated organizations, or those of the publisher, the editors and the reviewers. Any product that may be evaluated in this article, or claim that may be made by its manufacturer, is not guaranteed or endorsed by the publisher.
